# inSARa: intuitive single-target (large-scale) SAR interpretation and multi-target cross-reactivity analysis

**DOI:** 10.1186/1758-2946-6-S1-O18

**Published:** 2014-03-11

**Authors:** Sabrina Wollenhaupt, Knut Baumann

**Affiliations:** 1 Institut für Medizinische und Pharmazeutische Chemie, Technische Universität Braunschweig, Beethovenstr. 55, 38106 Braunschweig, Germany

## 

inSARa (**i**ntuitive **n**etworks for **S**tructure-**A**ctivity-**R**elationships **a**nalysis) was primarily developed with the objective to support the medicinal chemist in tackling SAR analysis and visualization of large data sets in a more intuitive way than fingerprint-based approaches [1]. The method takes advantage of the synergic combination of the reduced graph (RG) and the maximum common substructure (MCS) concept [2].

The main feature of the inSARa concept is a hierarchical network structure of clearly-defined substructure relationships based on common pharmacophoric features. Thus, straightforward SAR interpretation is possible by interactive network navigation. When focusing on a set of active molecules at one single target, the resulting inSARa networks were shown to be valuable for various essential tasks in SAR analysis, such as the identification of activity cliffs or activity switches, bioisosteric replacements or SAR hotspots. Based on the identification of nearest neighbours in the networks, the prediction of bioactivities is also possible.

Furthermore, inSARa can be used to investigate similarities between different targets. Targets are compared based on the overlap of common pharmacophoric pattern (RG MCSs) of the corresponding inSARa networks. According to the similar property principle, similar ligands are expected to bind to similar targets [3]. Therefore, this ligand-based analysis not only revealed meaningful similarity relationships between the analysed targets but is also beneficial for the detection of potential off-target relationships and cross-reactivities. Especially when investigating targets where no structural information is available but a set of active ligands is known (e.g. GPCRs), this complementary approach can provide important knowledge for drug design.
